# Increased Threat of Urban Malaria from *Anopheles stephensi* Mosquitoes, Africa 

**DOI:** 10.3201/eid2507.190301

**Published:** 2019-07

**Authors:** Willlem Takken, Steve Lindsay

**Affiliations:** Wageningen University & Research, Wageningen, the Netherlands (W. Takken);; Durham University, Durham, UK (S. Lindsay)

**Keywords:** *Anopheles stephensi*, Africa, urban malaria, mosquitoes, vectors, parasites, vector-borne infections

## Abstract

Malaria continues to be a major health threat in Africa, mainly in rural areas. Recently, the urban malaria vector *Anopheles stephensi* invaded Djibouti and Ethiopia, potentially spreading to other areas of Africa. Urgent action is needed to prevent urban malaria epidemics from emerging and causing a public health disaster.

The pernicious life-threatening disease malaria continues to place a heavy burden on communities in Africa, where >92% of malaria cases occur today (*1*). Mosquitoes of the genus *Anopheles* transmit malaria parasites to humans. Africa has >128 indigenous *Anopheles* species (*2*), several of which, *An. gambiae sensu stricto*, *An. coluzzii*, and *An. funestus sensu stricto*, are among the world’s most efficient malaria vectors. These species are found predominantly in rural areas, where they thrive in a variety of natural and manmade aquatic sites. Because mosquito densities fluctuate with rainfall, malaria is prevalent in rural areas in Africa with strong seasonal variations (*3*). 

Malaria also occurs in urban centers in Africa, but at much lower levels, mostly in the peripheries, where small-scale commercial gardens collect surface water (*4*). Malaria is not the only mosquito-borne disease threat in urban Africa. The *Aedes aegypti* mosquito is a vector for dengue, yellow fever, chikungunya, and Zika viruses in urban settings.

Many countries in Africa are experiencing rapid urban development because people from the countryside, attracted by opportunities for work and education, are moving into urban centers. According to the United Nations, cities like Nairobi, Kenya; Dar es Salaam, Tanzania; Kinshasa, Democratic Republic of the Congo; Lagos, Nigeria; Abidjan, Côte d'Ivoire; and Dakar, Senegal, have doubled in population during the last decade and are predicted to expand further (https://population.un.org/wup).

The global malaria eradication campaign, launched in 2005, has led to major reductions in malaria prevalence (*5*), but recent data on malaria in Africa suggest that further reductions are less clear. In many parts of sub-Saharan Africa, progress in malaria control has stalled, and malaria is still widespread (*1*). In addition, the campaign does not focus on urban areas, where malaria prevalence is low or absent.

In 2016, *An. stephensi* mosquitoes were found for the first time in Ethiopia, where this species has since become established (*6*). This discovery followed earlier reports of the species in neighboring Djibouti (*7*). *An. stephensi* mosquitoes are native to southern and western Asia, where the species serves as an efficient malaria vector (*8*). Unlike other malaria vectors in Africa, *An. stephensi* mosquitoes are found not only in rural areas but also in cities, where they breed in manmade water containers, such as household water storage containers and garden reservoirs. The *An. stephensi* mosquito is considered to be the main malaria vector in urban centers in India and Pakistan (*8*). Recently, the species was recorded for the first time in Sri Lanka, demonstrating its ability to disperse across large bodies of water and establish successfully in new geographic regions (*9*).

Because Africa currently does not have a malaria vector adapted to urban centers, establishment of *An. stephensi* mosquitoes on the continent poses considerable health risks. If the species disperses beyond its current distribution in eastern Ethiopia and successfully invades large cities, such as Khartoum, Sudan; Mombasa, Kenya; and Dar es Salaam, the region could face malaria outbreaks of unprecedented size. Because of relatively high levels of malaria prevalence in persons of all ages in rural areas, high mobility between rural and urban areas, and inadequate healthcare, countries in Africa are unprepared to deal with rapid spread of malaria in their cities and towns by a vector species well adapted to urban infrastructures. 

To halt the potential risk and prevent further spread of this vector requires urgent action. Historic examples demonstrate that a well-coordinated eradication of a species is possible, such as elimination of invasive *An. gambiae* mosquitoes from Brazil, as well as their eradication from Egypt. However, once a species disperses and covers larger geographic areas, eradication becomes nearly impossible. For example, the *Ae. albopictus* mosquito, a vector of chikungunya and dengue, has spread globally from its original location in Southeast Asia and has become a threat in many countries. 

The World Health Organization’s Global Vector Control Response 2017–2030 (GVCR; https://www.who.int/vector-control/publications/global-control-response/en) calls for multisectoral approaches to vector control. Urban mosquito control programs in Africa can use GVCR strategies to closely examine mosquito vectors thriving in cities and develop programs to reduce the threat to public health. In our view, surveillance for mosquito vectors in urban centers is essential for preventing outbreaks of infectious vectorborne diseases by eliminating newly established foci of vectors while they are still small (*10*). The invasion of *An. stephensi* mosquitoes on the African continent is a threat to health in tropical Africa but also provides an opportunity to build out vector control strategies as outlined in the GVCR.
